# PD-L1 expression in cerebrospinal fluid for leptomeningeal metastasis from solid tumors: preliminary assessment of clinical implications

**DOI:** 10.3389/fimmu.2025.1681280

**Published:** 2025-09-29

**Authors:** Zhenyu Pan, Hang Lei, Yushan Huang, Min Liu, Miaomiao Liu, Panpan Tai, Xiao Chen, Yu Wu, Lishi Wang, Yuancheng Zhang, Guozi Yang

**Affiliations:** Department of Radiation Oncology, The Affiliated Huizhou Hospital, Guangzhou Medical University, Huizhou, China

**Keywords:** leptomeningeal metastasis, PD-L1, cerebrospinal fluid, ThinPrep liquid-based cytology, immunotherapy

## Abstract

**Background:**

Intrathecal immune checkpoint inhibitors have shown promise for leptomeningeal metastasis (LM). Programmed cell death ligand 1 (PD-L1) is currently the primary predictive biomarker for immunotherapy response. This study assessed the feasibility of PD-L1 detection via immunocytochemistry based on ThinPrep liquid-based cytology (LBC), explored its application in cerebrospinal fluid (CSF) from LM patients and preliminarily evaluated clinical implications.

**Methods:**

Technical validation used six human tumor cell lines (lung, breast and gastric) with validated high/low PD-L1 expression, which were processed into ThinPrep LBC slides and cell block sections. PD-L1 immunocytochemistry and immunohistochemistry were performed using four antibodies (Dako 22C3, Ventana SP263, Abcam 28–8 and SP142). Clinically, CSF samples from LM patients were ThinPrep-processed and PD-L1 immunocytochemistry-stained. PD-L1 expression was assessed via tumor proportion score. Concordance of different antibody assays, as well as that of PD-L1 expression between paired CSF and extracranial lesions, was analyzed using Cohen’s κ. The association between CSF PD-L1 expression and response to intrathecal immunotherapy was assessed.

**Results:**

Immunocytochemistry based on ThinPrep LBC reliably detected membrane PD-L1, showing high concordance with immunohistochemistry on cell blocks for clones 22C3/SP263 across all cell lines (NCI-H358, MDA-MB-231, SGC-7901, A549, MCF7, and AGS). Clones 28-8/SP142 showed false positives and were excluded. Using clones 22C3/SP263, 130 CSF samples from 65 LM patients (55 lung, 8 breast, and 2 gastric cancers) were analyzed. Overall PD-L1 positivity rates were 48% with 22C3 and 51% with SP263 (κ=0.815). In the subset of 68 slides containing ≥100 tumor cells, PD-L1 positivity rates were 53% with 22C3 and 59% with SP263 (κ=0.881). For 62 slides containing 20–100 tumor cells, the rates were 42% (13/31) with both antibodies (κ=0.735). PD-L1 expression showed poor agreement between 29 paired CSF and extracranial lesions (κ=0.175 with 22C3; κ=0.179 with SP263). Among 45 patients receiving intrathecal immunotherapy, A numerical increase in response rate was observed in PD-L1-positive patients (61.9% vs. 33.3%; p=0.055).

**Conclusions:**

Our study establishes a robust methodology for detecting CSF PD-L1 expression using ThinPrep LBC with immunocytochemistry for LM patients. This approach suggests potential utility of CSF PD-L1 expression as a biomarker for guiding intrathecal immunotherapy for LM from solid tumors.

## 1 Introduction

Leptomeningeal metastasis (LM) is characterized by tumor cell dissemination within the leptomeninges and subarachnoid space. As a fatal complication of solid malignancies, LM leads to diffuse involvement of the entire central nervous system (CNS). The advent of immune checkpoint inhibitors has revolutionized oncology practice, with immunotherapy being increasingly applied in clinical cancer treatment and significantly improving outcomes for various tumor types, including lung cancer, head and neck cancer and many other malignancies (1–3). Recent studies have explored the use of programmed cell death protein 1 (PD-1) inhibitors in treating LM from solid tumors (4–6). Notably, a pioneering study demonstrated preliminary clinical efficacy of intrathecal PD-1 inhibitor administration in LM with melanoma (7). We are also conducting a series of clinical trials investigating combined intrathecal PD-1 inhibitors and chemotherapy for LM from solid tumors (ClinicalTrials.gov identifiers: NCT06462222, NCT06809530, NCT06809517, and NCT06762080).

Programmed cell death ligand 1 (PD-L1) expression serves as a cornerstone biomarker for PD-1 inhibitor therapy. While immunohistochemistry (IHC) on tissue specimens is the gold standard for PD-L1 assessment (8), it is not feasible in LM patients due to the inaccessibility of leptomeningeal tissue. Cerebrospinal fluid (CSF) provides a viable alternative samples for LM patients. Immunocytochemistry (ICC) analysis based on liquid samples enables direct visualization of the localization and intensity of target protein expression at the single-cell level. Recent studies have explored the feasibility of PD-L1 detection by ICC in malignant pleural fluid samples using centrifuged cell block preparations (9, 10). However, for LM patients, CSF samples contain significantly fewer tumor cells than those found in malignant pleural effusion or ascites, resulting in an inherent false-negative rate for CSF cytology (11). Furthermore, the volume of CSF obtainable for testing per single collection is severely limited. These constraints make it impossible to perform conventional centrifugation-based cell block preparation and analysis on CSF samples. To date, research on PD-L1 detection in CSF has been restricted to enzyme-linked immunosorbent assay (ELISA)-based quantification of soluble PD-L1 (sPD-L1) levels, which has shown diagnostic and prognostic value in primary CNS lymphoma and glioma (12, 13). No studies have reported PD-L1 ICC on CSF samples, highlighting a gap in immunotherapy-related biomarker research for LM.

The ThinPrep liquid-based cytology (LBC) platform offers improved cellular enrichment and preservation of membrane morphology compared to conventional cytospin techniques. Our previous work demonstrated its superior diagnostic performance in CSF cytology for LM (14). Based on our previous findings, this study evaluated the feasibility of PD-L1 detection via ICC based on ThinPrep LBC and assessed its application in CSF from LM patients. Furthermore, exploratory analyses were conducted to investigate potential correlations between CSF PD-L1 expression and clinical responses to intrathecal immunotherapy in this patient cohort.

## 2 Materials and methods

### 2.1 Cell lines and processing

To validate the feasibility of PD-L1 ICC analysis using ThinPrep LBC, six human cell lines with confirmed high/low PD-L1 expression were selected as PD-L1 positive/negative controls: lung adenocarcinoma (NCI-H358 [PD-L1-high] vs. A549 [PD-L1-low]), breast cancer (MDA-MB-231 [PD-L1-high] vs. MCF7 [PD-L1-low]), and gastric adenocarcinoma (SGC-7901 [PD-L1-high] vs. AGS [PD-L1-low]). These cell lines were obtained from the Cell Bank of the Chinese Academy of Sciences and cultured in RPMI-1640 Medium (Cat# C11875500BT, Gibco, USA) or Dulbecco’s Modified Eagle’s Medium (DMEM; Cat# C11965500BT, Gibco, USA), supplemented with 10% fetal bovine serum (Cat# A5256701, Gibco, USA), 100 U/ml penicillin, and 100 μg/ml streptomycin (Cat# 15140122, Gibco, USA). Cultures were maintained at 37°C with 5% CO_2_ in a humidified incubator.

Samples of each cell line were processed into ThinPrep LBC slides using the ThinPrep 2000 automated slide processor (Hologic, Bedford, MA, USA) following the manufacturer’s instructions. In addition, cell samples from NCI-H358, MDA-MB-231, and SGC-7901 were fixed in 4% paraformaldehyde (Cat# DF0135, Leagene, CN) for one hour, then embedded in agarose (Cat# BS081, Biosharp, CN) using a fully enclosed tissue processor (Leica ASP300S, Wetzlar, DE). Subsequently, 4-μm-thick sections were prepared from these cell blocks.

### 2.2 Patient selection

CSF samples in this study were from LM patients enrolled in the following clinical trials: NCT06304441, NCT06462222, NCT06809530, NCT06809517, and NCT06762080. Enrolled patients must meet the following inclusion criteria: (1) confirmed histological diagnosis of solid tumors; (2) positive CSF cytology; and (3) ≥20 tumor cells on ThinPrep LBC slides. Patient demographics, clinicopathological information, PD-L1 expression status of extracranial lesions and treatment response data were collected through retrospective manual electronic health record review. The study received approval from the Institutional Ethics Committee of the Affiliated Huizhou Hospital, Guangzhou Medical University and was conducted in accordance with the principles of the Declaration of Helsinki. Written informed consents were obtained from all participants, indicating their willingness to donate their CSF samples for research. Permission to access and publish patient information was also obtained from each patient.

### 2.3 Assessment of treatment response and follow-up

Intrathecal pharmacotherapy (including immunotherapy and chemotherapy) was given to enrolled patients. Follow-up time is defined as the period from enrollment until death or July 31, 2025, whichever comes first. The treatment response was evaluated by investigators blinded to clinical data of patients according to Response Assessment in Neuro-Oncology (RANO)-LM working group proposal criteria (15), which was based on three levels of assessment, CSF cytology, neuroimaging findings and clinical evaluation. Clinical response was defined as at least one of the evaluations of CSF cytology, neurological status and neuroimaging findings rated as improved, and there was no disease worsening at the same time. Stable disease was interpreted as a stable neurological examination, stable or equivocally worsening or improved neuroimaging findings and stable CSF cytology. Disease progression was defined as the worsening of neuroimaging or neurological dysfunction.

### 2.4 CSF sample collection and processing

CSF sample (18–20 mL) from each patient was collected by lumbar puncture or Ommaya reservoir before intrathecal pharmacotherapy, then added to PreservCyt cell preservation solution, and mixed. Slides were prepared using the ThinPrep 2000 automated slide processor (Hologic, Bedford, MA, USA) following the manufacturer’s instructions.

### 2.5 PD-L1 immunocytochemistry/immunohistochemistry

Following ThinPrep LBC slide preparation, samples were immediately fixed in 95% ethanol to preserve cellular morphology. PD‐L1 ICC staining was performed on ThinPrep LBC slides prepared from cell line suspensions using four anti-PD-L1 clones: two clinically validated anti-PD-L1 clones (SP263 [Ventana Medical Systems, USA] and 22C3 [Agilent Technologies, USA]) and two research-use-only PD-L1 antibodies (28–8 [Cat# ab205921, Abcam, UK] and SP142 [Cat# ab228462, Abcam, UK]). Subsequently, available PD-L1 antibodies were used for detecting PD-L1 expression in CSF samples. PD-L1 IHC was conducted on cell block sections from the tumor cell lines, using a modified protocol with 4% paraformaldehyde fixation, consistent with the ICC methodology.

### 2.6 Interpretation of PD‐L1 staining

Immunostained slides were evaluated by 5 independent pathologists. PD-L1 expression was quantified using the tumor proportion score (TPS), defined as the percentage of viable tumor cells with membranous PD-L1 staining relative to total tumor cells. Conventional TPS evaluation of PD-L1 requires ≥100 evaluable tumor cells per slide (per College of American Pathologists guidelines); however, given the low cellularity of CSF, this study expanded the inclusion criteria to accept CSF samples with 20–100 tumor cells to enhance clinical applicability. For slides with ≥100 tumor cells, multiple representative microscopic fields were systematically evaluated to enhance the accuracy of TPS quantification. For slides with 20–100 tumor cells, the absolute counts of PD-L1-positive tumor cells were recorded. TPS was calculated as the percentage of viable tumor cells with PD-L1 expression among at least 20 cells, and defined as positive when TPS ≥1%. The results were analyzed using predefined categorical thresholds: < 1%, 1-49% and ≥ 50%.

### 2.7 Statistical analysis

IBM SPSS Statistics 23.0 and GraphPad Prism 9.0 were used for all statistical analyses and *p*-values below 0.05 were considered significant. Inter-antibody concordance in PD-L1 scoring was quantitatively evaluated through Cohen’s κ coefficient analysis, with interpretation guided by Landis and Koch’s benchmark criteria (κ values: 0.21-0.40=fair; 0.41-0.60=moderate; 0.61-0.80=substantial; 0.81-1.00=near-perfect agreement). McNemar’s test was used to determine whether a statistically significant difference existed between the results of the two PD-L1 antibodies (SP263 and 22C3) detection. Pearson’s chi-squared test and Fisher’s exact test were employed to assess the association between CSF PD-L1 expression and patients’ clinical characteristics and disease-related variables. Graphics were generated using Bland–Altman plots to compare matched CSF and extracranial lesion samples.

## 3 Results

### 3.1 The feasibility of PD-L1 immunocytochemistry using ThinPrep liquid-based cytology

**Figure 1:**
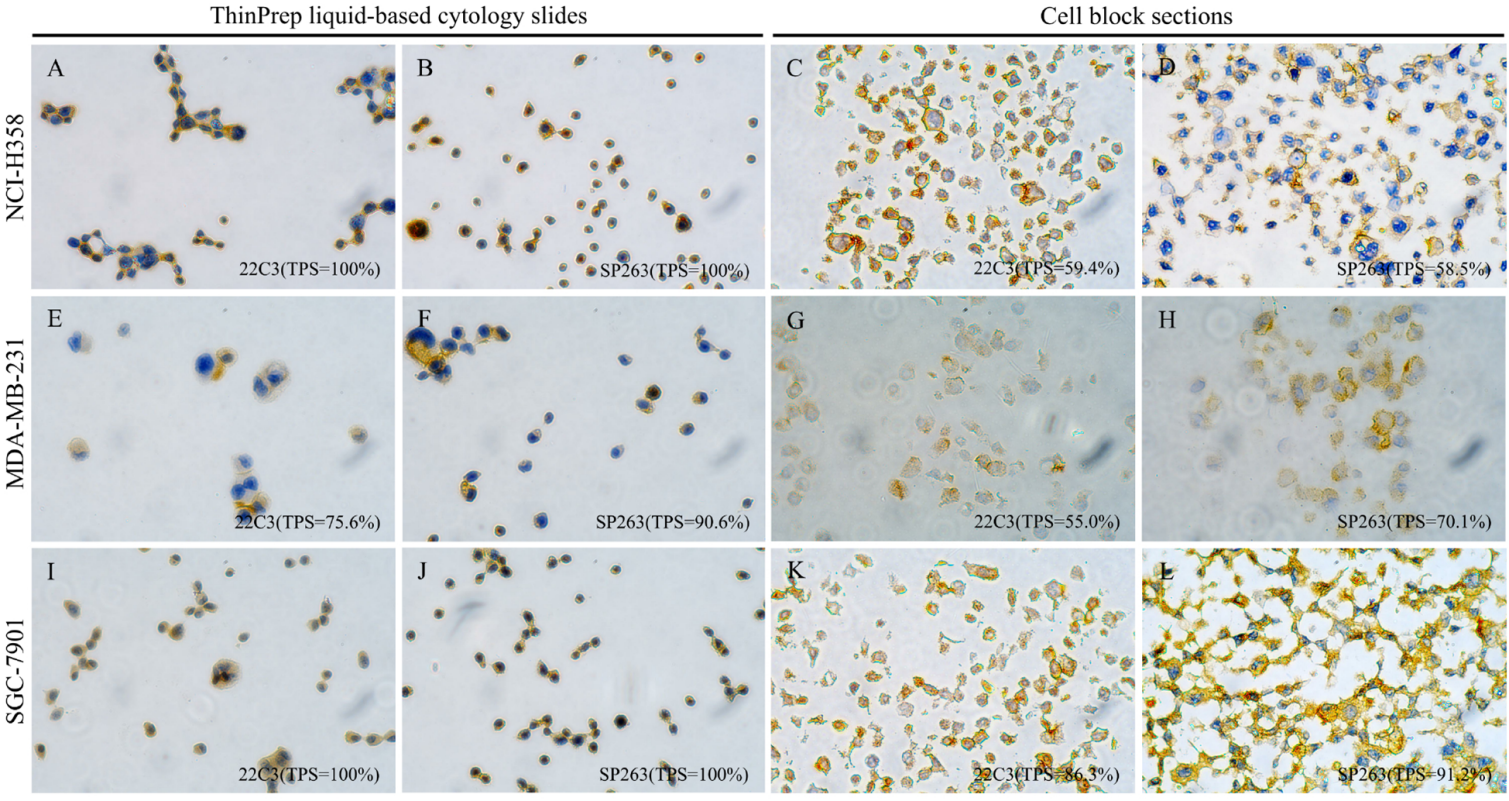
PD-L1 immunostaining with clones 22C3 (Dako) and SP263 (Ventana) on matched ThinPrep liquid-based cytology slides and cell block sections from three validated high-PD-L1-expression tumor cell lines (lung adenocarcinoma NCI-H358, breast cancer MDA-MB-231, and gastric cancer SGC-7901) (×400). Left panel **(A, B, E, F, I, J)**: Immunocytochemistry on ThinPrep liquid-based cytology slides displays PD-L1 staining manifesting as a circumferential ring-like pattern that enveloping the entire cell; Right panel **(C, D, G, H, K, L)**: Immunohistochemistry on paired cell block sections reveals distinct and crisp membranous PD-L1 staining.

As shown in **Figure 1** (left panel), in tumor cell lines with high PD-L1 gene expression, PD-L1 ICC using ThinPrep LBC yielded the following TPS: NCI-H358 (22C3: TPS = 100%; SP263: TPS = 100%), MDA-MB-231 (22C3: TPS = 75.6%; SP263: TPS = 90.6%), and SGC-7901 (22C3: TPS = 100%; SP263: TPS = 100%). Parallel PD-L1 IHC on cell block sections from the same cell lines showed TPS of 59.4% with 22C3 and 58.5% with SP263 in NCI-H358, 55% with 22C3 and 70.1% with SP263 in MDA-MB-231, and 86.3% with 22C3 and 91.2% with SP263 in SGC-7901 (**Figure 1**, right panel). In contrast, cell lines with low PD-L1 gene expression (A549, MCF7, and AGS) demonstrated minimal PD-L1 expression (TPS <1%) with both 22C3 and SP263 assays on ThinPrep LBC slides (**Supplementary Figure S1**). The other two research-use-only PD-L1 antibodies (28–8 and SP142, Abcam) were not available for the following study because they produced false-positive staining in the low-PD-L1-expressing MCF7 cell line (**Supplementary Figure S2**).

Notably, PD-L1 staining on ThinPrep LBC slides manifested as a circumferential ring-like pattern that enveloped the entire cell, which was fully different from membrane PD-L1 staining on tissue sections (**Figure 1**). To confirm that the PD-L1 expression detected on ThinPrep LBC slides accurately represented genuine membranous localization, we performed parallel PD-L1 IHC on cell block sections prepared from the same cell lines. As shown in **Figure 1**, cell lines with high PD-L1 expression displayed distinct and crisp membranous PD-L1 staining on cell block sections, while minimal or no membranous staining was observed in low-expressing cell lines. These findings were consistent with the staining patterns observed on the corresponding ThinPrep LBC slides. Collectively, these results demonstrated the feasibility of PD-L1 ICC using ThinPrep LBC and confirmed that PD-L1 expression detected on ThinPrep LBC slides represented authentic membranous staining on tumor cells.

### 3.2 Patient characteristics

Between July 2024 and July 2025, a total of 93 patients were screened and 65 LM patients (17 males and 48 females, median age: 54) were enrolled. The remaining 28 patients were excluded due to insufficient CSF cell counts. All enrolled patients were diagnosed with LM by positive CSF cytology. The primary tumor types included lung adenocarcinoma (n=55), breast cancer (n=8), and gastric adenocarcinoma (n=2). Among these patients, 29 had paired extracranial lesion samples, and 45 received intrathecal immunotherapy. Univariate analysis using the Pearson’s chi-squared and Fisher’s exact test revealed that PD-L1 expression in the CSF of LM patients was significantly associated with the primary tumor type of lung adenocarcinoma (22C3 assay: *p* = 0.028; SP263 assay: *p* = 0.008) and EGFR mutation (both 22C3 and SP263 assays: *p* = 0.049). Patients’ baseline clinical characteristics and disease-related variables are displayed in **Table 1** and **Supplementary Table S1**.

**Table 1:** The relationship between the expression of PD-L1 and the clinicopathological features.

Clinicopathological factors	n	PD-L1 expression^b^	
		22C3	SP263
Gender			
Male	17 (26)	0.938	0.180
Female	48 (74)		
Median age (years)	54		
KPS			
<60	25 (38)	0.287	0.239
≥60	40 (62)		
CSF cytology positive	65 (100)		
Neuroimaging features			
Yes	58 (89)	0.428	0.424
No	7 (11)		
Pathological types of primary disease			
Lung adenocarcinoma	55 (85)	0.028	0.008
Breast cancer	8 (12)	0.056	0.044
Gastric adenocarcinoma	2 (3)	0.028	0.253
Elevated intracranial pressure			
Yes	33 (51)	0.375	0.045
No	24 (37)		
Not evaluable	8 (12)		
Lung cancer			
EGFR	44 (68)	0.049	0.049
ALK	2 (3)	0.545	0.511
ROS	2 (3)	0.545	0.511
Others^a^	7 (11)		
Breast cancer			
Triple-negative	5 (8)		
Her-2 low expression	2 (3)		
Luminal A	1 (2)		
Brain metastasis			
Yes	36 (55)	0.529	0.853
No	29 (45)		
Prior treatment			
Prior molecular targeted treatment	57 (88)		
Prior systemic chemotherapy	38 (58)		
Prior immunotherapy	16 (25)		
Paired extracranial lesions			
Yes	29 (45)		
No	36 (55)		
Intrathecal immunotherapy			
Yes	45 (69)		
No	20 (31)		

a Including 1 case with RET mutation, 1 case with KRAS (G12C) mutation, 1 case with Her2 (3+), 3 cases with no sensitive mutation, and 1 not detected.

b Pearson's chi-squared test and Fisher's exact test were employed to assess the association between CSF PD-L1 expression and patient’s clinical characteristics and disease-related variables. *p*-values below 0.05 were considered significant.

PD-L1, Programmed cell death ligand 1; KPS, Karnofsky Performance Status; CSF, Cerebrospinal fluid; EGFR, Epidermal Growth Factor Receptor; ALK, Anaplastic Lymphoma Kinase; ROS, Reactive Oxygen Species.

### 3.3 The feasibility of PD-L1 assessment in CSF from LM patients

A total of 130 CSF samples were collected from 65 patients, comprising 68 samples with ≥ 100 tumor cells and 62 samples with 20–100 tumor cells (**Table 2**). Overall PD-L1 positivity (TPS ≥1%) was numerically lower with the 22C3 than with SP263 (31/65 [48%] vs. 33/65 [51%]; *p* = 0.727). Among PD-L1-positive samples, the majority exhibited low to moderate PD-L1 expression (TPS 1-49%), accounting for 94% (29/31) with 22C3 and 97% (32/33) with SP263.

**Table 2:** Detailed concordance of PD-L1 expression in CSF between 22C3 and SP263 assay.

Variable	22C3 assay	SP263 assay	Cohen's κ
130 CSF specimens from 65 LM patients, n (%)			
TPS <1%	34(52)	32(49)	0.815
TPS = 1-49%	29(45)	32(49)	
TPS ≥50%	2(3)	1(2)	
68 CSF specimens with ≥ 100 tumor cells, n (%)			
TPS <1%	16(47)	14(41)	0.881
TPS = 1-49%	17(50)	20(59)	
TPS ≥50%	1(3)	0(0)	
62 CSF specimens with <100 tumor cells, n (%)			
TPS <1%	18(58)	18(58)	0.735
TPS = 1-49%	12(39)	12(39)	
TPS ≥50%	1(3)	1(3)	
110 CSF specimens from 55 lung adenocarcinomas, n (%)			
TPS ≥1%	30(55)	32(58)	0.778
16 CSF specimens from 8 breast cancers, n (%)			
TPS ≥1%	1(13)	1(13)	
4 CSF specimens from 2 gastric adenocarcinomas, n (%)			
TPS ≥1%	0(0)	0(0)	

PD-L1, Programmed cell death ligand 1; CSF, Cerebrospinal fluid; LM, Leptomeningeal metastasis; TPS, Tumor proportion score.

**Figure 2:**
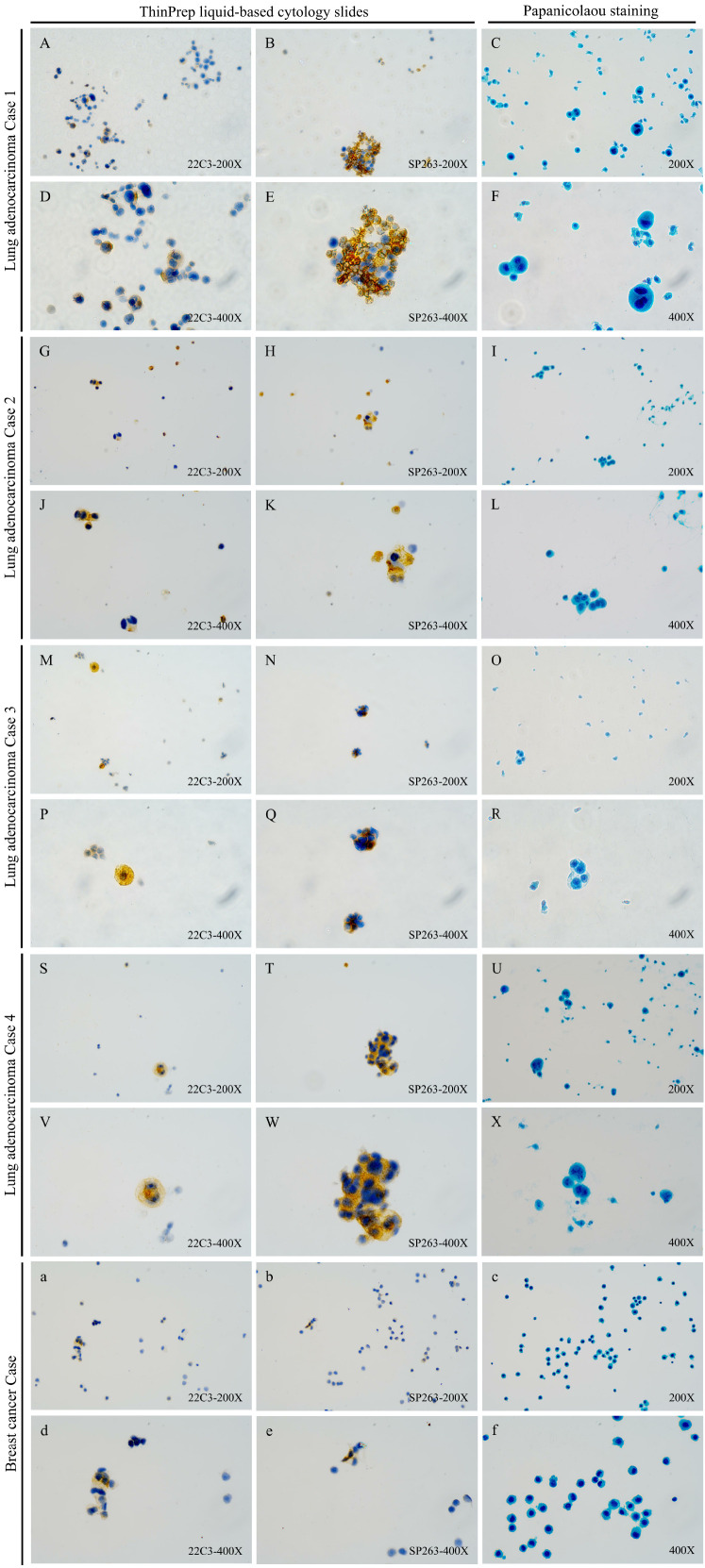
PD-L1 immunocytochemistry with clones 22C3 (Dako) and SP263 (Ventana) on CSF ThinPrep liquid-based cytology slides from LM patients with Lung adenocarcinoma or breast cancer: representatives of different TPS in slides with different tumor cell number stratification (×200 & ×400). **(A–F)** Detection results of the 22C3 (TPS:10.0%) and SP263 (TPS:12.0%) antibodies and papanicolaou staining in a PD-L1-positive lung adenocarcinoma case 1 (≥100 tumor cells); **(G–L)** Detection results of the 22C3 (TPS:59.2%) and SP263 (TPS:40.6%) antibodies and papanicolaou staining in a PD-L1-positive lung adenocarcinoma case 2 (≥100 tumor cells); **(M–R)** Detection results of the 22C3 (TPS:2.8%) and SP263 (TPS:16.7%) antibodies and papanicolaou staining in a PD-L1-positive lung adenocarcinoma case 3 (20–100 tumor cells); **(S–X)** Detection results of the 22C3 (TPS:75.0%) and SP263 (TPS:88.0%) antibodies and papanicolaou staining in a PD-L1-positive lung adenocarcinoma case 4 (20–100 tumor cells); **(a–f)** Detection results of the 22C3 (TPS:8.6%) and SP263 (TPS:3.5%) antibodies and papanicolaou staining in a PD-L1-positive breast cancer case (≥100 tumor cells).

As shown in **Table 2**, when samples were stratified by CSF tumor cellularity (≥100 vs. 20–100 tumor cells), a consistent trend was observed in the subgroup with ≥100 tumor cells (n=68), where 22C3 demonstrated lower PD-L1 positivity than SP263 (18/34 [53%] vs. 20/34 [59%]; *p* = 0.624). However, in the subgroup with 20–100 tumor cells (n=62), both 22C3 and SP263 exhibited similar positivity rates (13/31 [42%] for both; *p* = 1.000). Neither comparison reached statistical significance. Moreover, high inter-assay concordance (Cohen’s κ) was observed between the two antibodies at the ≥1% TPS threshold across all cohorts: overall concordance in 130 samples (κ=0.815), in samples with ≥100 tumor cells (κ=0.881), and in samples with 20–100 tumor cells (κ=0.735). Similarly, strong concordance was observed within the LM from lung adenocarcinoma subgroup (κ= 0.778, **Figure 2**). Notably, PD-L1 expression in the CSF was negative in the two LM patients with gastric cancer (**Supplementary Figure S3**); among eight LM patients with breast cancer, only one showed positive PD-L1 expression in CSF (**Figure 2**). These findings may be attributed to the limited sample size.

### 3.4 Concordance of PD-L1 expression between paired CSF and extracranial lesion

**Figure 3:**
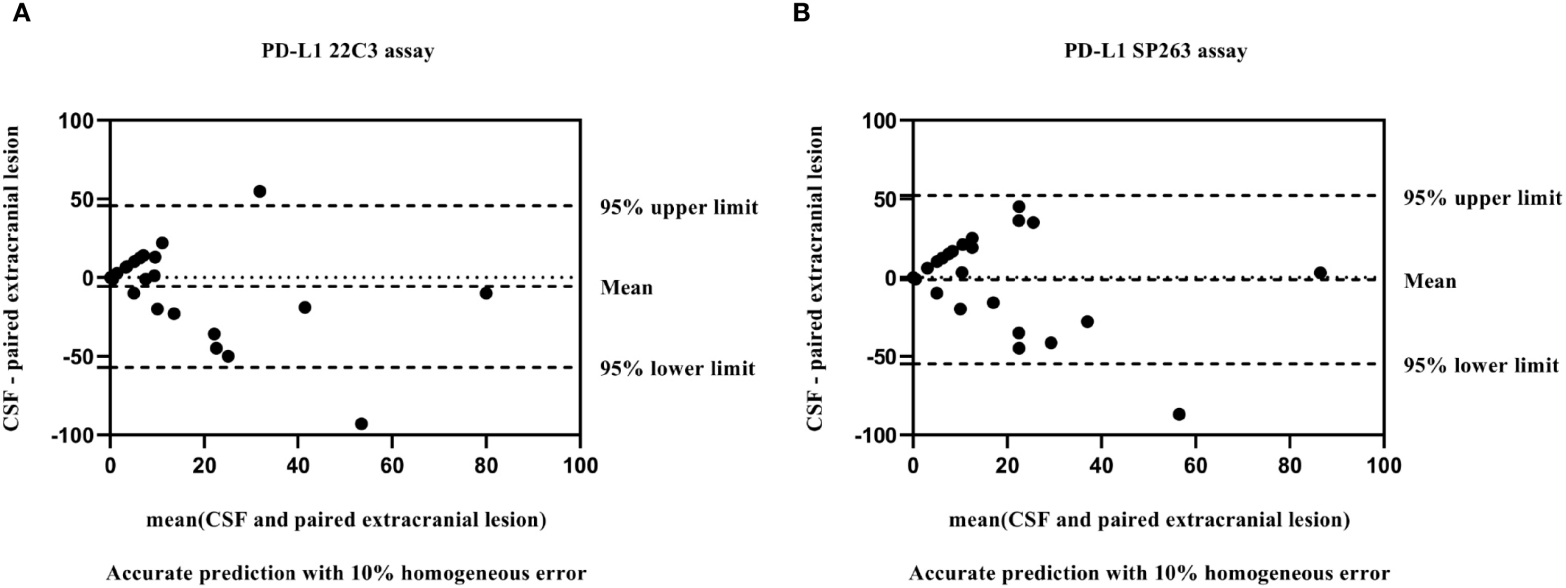
Bland–Altman plot: comparison of PD-L1 values between matched CSF and extracranial lesion samples with 22C3 **(A)** and SP263 assays **(B)**.

A total of 29 patients had paired extracranial lesion samples, including 24 primary tumor tissues and 5 metastatic lesion samples. The samples showed 15 with TPS<1%, 10 with TPS 1-49% and 4 with TPS≥50%. When we evaluated the consistency of PD-L1 expression between CSF and extracranial lesion samples using Bland–Altman plot, we observed a slight underestimation of PD-L1 expression in CSF samples compared with extracranial lesion samples, regardless of whether 22C3 or SP263 assays were used (**Figure 3**). In addition, as detailed in **Table 3**, we found a poor consistency between extracranial lesions and CSF samples (κ=0.175 with 22C3 assay; κ=0.179 with SP263).

**Table 3:** Detailed concordance of PD-L1 expression between CSF and extracranial lesion.

Extracranial lesion (n=29)	CSF with 22C3 assay (n=29)			CSF with SP263 assay (n=29)		
	TPS<1%	1%≤ TPS <50%	TPS≥50%	TPS<1%	1%≤ TPS <50%	TPS≥50%
TPS<1%	8	7	0	7	8	0
1%≤ TPS <50%	4	5	1	4	6	0
TPS≥50%	1	2	1	0	3	1

PD-L1, Programmed cell death ligand 1; CSF, Cerebrospinal fluid; TPS, Tumor proportion score.

### 3.5 Association of CSF PD-L1 expression with response to intrathecal immunotherapy

Among the 65 enrolled patients, 62 underwent intrathecal pharmacotherapy (45 receiving intrathecal immunotherapy and 17 receiving intrathecal chemotherapy), while 3 patients declined LM-related treatment due to personal reasons. Using the SP263 assay, PD-L1 expression in CSF was positive (TPS ≥1%) in 31 patients and negative (TPS <1%) in the remaining 31. From July 2024 to July 31, 2025, the median follow-up for all enrolled patients was 4.5 months (range 1.1-17.8 months). The clinical response rate was numerically higher in PD-L1-positive patients (54.8%, 17/31) compared to PD-L1-negative patients (32.3%, 10/31; *p* = 0.073; **Supplementary Table S2**). In the intrathecal immunotherapy subgroup (n=45), PD-L1 expression was positive in 21 patients and negative in 24 patients. Clinical outcomes comprised 21 patients with clinical response, 21 with stable disease, and 3 with progressive disease. A numerical increase in clinical response rate was observed in PD-L1-positive patients (61.9% vs. 33.3%; *p* = 0.055, **Table 4**), representing an absolute difference of 28.6%. Multivariate analysis revealed that PD-L1 expression, along with other clinically relevant factors including primary tumor type, molecular characteristics, and prior treatments, did not exert significant influence on the clinical response in 45 LM patients receiving intrathecal immunotherapy (**Supplementary Figure S4**). However, this difference did not reach statistical significance, and due to the limited sample size, the results should be interpreted as exploratory. Similar numerical differences were observed using the 22C3 assay (**Table 4**, **Supplementary Table S2**). These findings require validation in larger cohorts to draw meaningful conclusions regarding the association between PD-L1 expression and treatment response. Additionally, due to insufficient follow-up duration, the correlation between CSF PD-L1 expression and longer-term clinical outcomes such as progression-free survival and overall survival remains under evaluation through extended monitoring.

**Table 4:** The relationship between CSF PD-L1 expression and response to intrathecal immunotherapy.

Response to intrathecal immunotherapy	CSF with 22C3 assay (n=45)		*P* value	CSF with SP263 assay (n=45)		*P* value
	TPS<1% (n = 24)	TPS≥1% (n = 21)		TPS<1% (n = 24)	TPS≥1% (n = 21)	
Clinical response, n (%)	8 (33)	13 (62)	0.055	8 (33)	13 (62)	0.055
Stable disease, n (%)	13 (54)	8 (38)		14 (58)	7 (33)	
Progressive disease, n (%)	3 (13)	0 (0)		2 (8)	1 (5)	

Stable disease and progressive disease were defined as no-response. The chi-square test was employed to assess the association between TPS group (</≥1%) and clinical response (clinical response vs. no-response).

PD-L1, Programmed cell death ligand 1; CSF, Cerebrospinal fluid; TPS, Tumor proportion score.

## 4 Discussion

Emerging clinical studies demonstrate the promising therapeutic efficacy of intrathecal immunotherapy in LM from melanoma (7, 15). Given that PD-L1 expression remains the only clinically validated predictive biomarker for immunotherapy response, its assessment in CSF holds significant translational value for LM management. This pilot study established a reliable PD-L1 detection method in CSF samples from LM patients with solid tumors, utilizing ThinPrep LBC combined with standardized ICC protocols. This methodological validation confirmed the feasibility of detecting PD-L1 expression in tumor cells from CSF samples and suggests potential utility of CSF PD-L1 as a biomarker for the application of intrathecal immunotherapy. Notably, our preliminary findings revealed that PD-L1 expression profiles in CSF varied significantly across primary tumor types. More importantly, exploratory analysis in a limited cohort observed discordance between PD-L1 expressions in CSF and matched extracranial lesion, suggesting that PD-L1 expression in CSF may not be reliably substituted by extracranial lesion. This discrepancy warrants further investigation into the potential immune heterogeneity between LM and systemic tumors.

This study first investigated the technical feasibility of PD-L1 detection using ThinPrep LBC. Comprehensive comparative analyses were conducted between ICC on ThinPrep LBC slides and IHC on cell block sections, covering multiple cancer cell lines (lung adenocarcinoma, breast cancer, and gastric cancer) with varying PD-L1 expression profiles. The results revealed excellent concordance between ICC and IHC when using clinically validated PD-L1 antibody clones 22C3 (Agilent/Dako) and SP263 (Ventana), accurately reflecting known cell line expression patterns. In contrast, clones 28-8 (abcam) and SP142 (abcam) exhibited discordant performance, with false-positive staining observed in PD-L1-low cell lines. These findings validated ICC on ThinPrep-processed slides using 22C3 or SP263 clones as a reliable and clinically applicable method for PD-L1 detection in CSF-derived tumor cells. This approach offers a practical solution for PD-L1 assessment in challenging paucicellular liquid biopsy specimens where traditional methods may be inadequate.

PD-L1 is a transmembrane protein primarily localized to the cell membrane. In IHC of sectioned tissues or cell blocks, PD-L1-positive cells typically exhibit distinct membrane-specific staining patterns. In contrast, ThinPrep cytology preserves the intact three-dimensional cellular architecture and maintains native cellular morphology by immobilizing whole cells onto slides without sectioning. Following PD-L1 immunostaining, positive cells exhibited circumferential membrane staining that appeared as diffuse whole-cell coloration under microscopic examination. This phenomenon results from the en face visualization of the intact cell membrane in three dimensions. To validate the membrane specificity of this staining pattern, parallel IHC analyses were performed on cell block sections derived from the same cell lines. Comparative analysis confirmed that both preparation methods reliably detect membrane-localized PD-L1 expression. Our findings demonstrated that while ThinPrep-based ICC generates phenotypically distinct staining patterns (diffuse whole-cell coloration) compared to the crisp linear membrane staining observed in conventional two-dimensional IHC sections, it maintains equivalent detection accuracy. This observation aligns with previously reported findings from ICC in pleural effusions samples using other cytological preparation methods (smears/cytospins) (16).

When assessing the concordance of the two antibodies in ICC testing, we found a strong agreement between 22C3 and SP263 assays across all CSF samples (κ=0.815). Similar concordance was observed in the subgroup of LM patients with lung adenocarcinoma (κ=0.778). These findings align with the results in the Blueprint phase I/II studies, where phase II real-world data revealed concordance rates of 86.4% and 91.5% between the SP263 and 22C3 assays at TPS thresholds of ≥1% and ≥50%, respectively (17, 18). In addition, SP263 assay in this study exhibited a marginally higher positivity rate (51%) compared to 22C3 assay (48%) and consistently generated higher TPS values than the 22C3 assay in nearly all positive CSF samples. This performance differential between the two PD-L1 assays was also consistent with previous Blueprint study findings (17, 18). While this discrepancy may reflect clone-specific epitope recognition or technical variables (e.g., antibody dilution ratios, DAB detection systems), the robust concordance at clinically relevant thresholds (TPS ≥1%) supports the reliability of both clones for assessment of PD-L1 expression in CSF from LM patients.

Accumulating evidence demonstrates significant correlations between tumor PD-L1 expression levels and improved clinical outcomes following immune checkpoint inhibitor therapy, including enhanced objective response rates and prolonged survival (19–23).While previous studies have employed ELISA to quantify sPD-L1 levels in CSF as a prognostic biomarker in primary CNS lymphoma and glioma (13, 24), the biological and clinical implications of sPD-L1 remain uncertain due to its heterogeneous origins (e.g., tumor-derived vs. immune cell-derived) and unclear functional significance (25). In contrast to ELISA-based detection of sPD-L1 in CSF, our approach directly evaluates membrane-bound PD-L1 expression on tumor cell surfaces, offering a more precise representation of the target relevant for immune checkpoint blockade therapy. Consequently, this methodology provides clinically actionable biomarkers for guiding immunotherapy decisions by enabling evaluation of tumor cell surface PD-L1 expression in CSF, demonstrating potential superior predictive performance compared to approaches relying exclusively on sPD-L1 measurements.

PD-L1 expression is typically lower in breast and gastric cancers than in lung cancer (26). In our study, we also observed differential PD-L1 expression patterns in CSF of LM patients based on primary tumor origin. Specifically, we observed a significantly higher PD-L1 positivity rate in CSF from LM patients with lung adenocarcinoma compared to those with breast or gastric cancer. This trend is consistent with the known heterogeneity of PD-L1 expression across cancer types. However, due to the limited sample size within these subgroups, these comparisons should be interpreted with caution and regarded as exploratory. Additionally, PD-L1 expression is dynamic over the course of disease. In our cohort of 29 patients with matched samples, we found discordant PD-L1 expression profiles between CSF and extracranial lesions, underscoring the unique immunological features of the CNS metastatic microenvironment compared to systemic tumors. These findings support the potential clinical relevance of CSF-based PD-L1 assessment, which may provide distinct and complementary information to conventional tissue-based testing. Further studies with larger cohorts are warranted to validate these preliminary observations.

Systemic immunotherapy faces significant challenges in crossing the blood-CSF barrier, resulting in poor clinical outcomes for LM, with low response rates and a median overall survival (mOS) of only 2.9–3.6 months (4, 5). Intrathecal PD-1 inhibitor has recently emerged as a feasible and safe strategy (15, 27, 28), with combined intrathecal and intravenous administration showing promise in melanoma-related LM (mOS 4.9 months) and a one-year survival rate of 26% (7). However, most solid tumors are immunotherapy-”cold,” necessitating combination strategies. We are currently conducting clinical trials on intrathecal immunotherapy combined with intrathecal chemotherapy for LM from solid tumors (NCT06462222, NCT06809530, NCT06809517, and NCT06762080). In this context, assessment of PD-L1 expression in CSF may provide valuable information for personalizing intrathecal immunotherapy in LM patients. In this exploratory analysis, patients with PD-L1-positive CSF showed a numerically higher clinical response rate than those with negative expression (61.9% vs. 33.3%, p=0.055). Although this difference did not reach statistical significance in our limited cohort, the observed result suggests CSF PD-L1 expression may have potential utility as a biomarker to guide intrathecal immunotherapy strategies in LM. It should be emphasized, however, that the current analysis is primarily focused on response evaluation, as the relatively short follow-up duration in this interim dataset limits the robust assessment of survival endpoints such as progression-free survival (PFS) and overall survival (OS). With extended follow-up, we anticipate that future analyses from our ongoing trials will include larger sample sizes and sufficient data maturity to evaluate the correlation between CSF PD-L1 status and long-term survival outcomes.

This study has several limitations. First, due to the low incidence of LM, this study has a limited sample size, particularly with respect to LM patients with breast or gastric cancer. Therefore, the findings should be interpreted with caution and regarded as exploratory. More definitive conclusions are expected to emerge from our ongoing trials, which involve larger patient cohorts. Second, LM typically develops years after the diagnosis of the primary tumor. The clinical challenge of obtaining matched extracranial lesion samples for LM patients often means that paired analyses are infrequent. Consequently, the available data are insufficient to definitively establish a relationship between PD-L1 expression in CSF-derived tumor cells and extracranial lesions. Third, the clinical implications of our findings are limited by the relatively short-term follow-up period after intrathecal immunotherapy. A longer observation period, coupled with systematic clinical data collection, is necessary to fully elucidate the prognostic significance of PD-L1 expression in the CSF of LM patients.

## 5 Conclusion

This study establishes a robust methodology for detecting PD-L1 expression in CSF-derived tumor cells using ThinPrep LBC with standardized ICC protocols, thereby validating CSF as a viable liquid biopsy source for LM patients. Our findings suggest preliminary evidence of discordance between PD-L1 status in CSF and matched extracranial lesions. This compartment-specific divergence may underscore the existence of distinct immune microenvironments in LM compared to systemic disease, potentially supporting the need for direct assessment of CSF biomarkers rather than reliance on peripheral samples. As PD-L1 remains the primary clinically validated predictor of immunotherapy response, our results may suggest potential utility of CSF PD-L1 expression as a biomarker for guiding intrathecal immunotherapy for LM from solid tumors.

## Data Availability

The original contributions presented in the study are included in the article/**Supplementary Material**. Further inquiries can be directed to the corresponding author.
