# Supplementary material for: PD-L1 expression in cerebrospinal fluid for leptomeningeal metastasis from solid tumors: preliminary assessment of clinical implications
Source: Front Immunol. 2025 Sep 29;16:1681280. doi: 10.3389/fimmu.2025.1681280 (PMC12515682; doi:10.3389/fimmu.2025.1681280)
Supplement: Supplementary file 2 [file DataSheet1.docx]

Supplementary Material

# Supplementary Figures


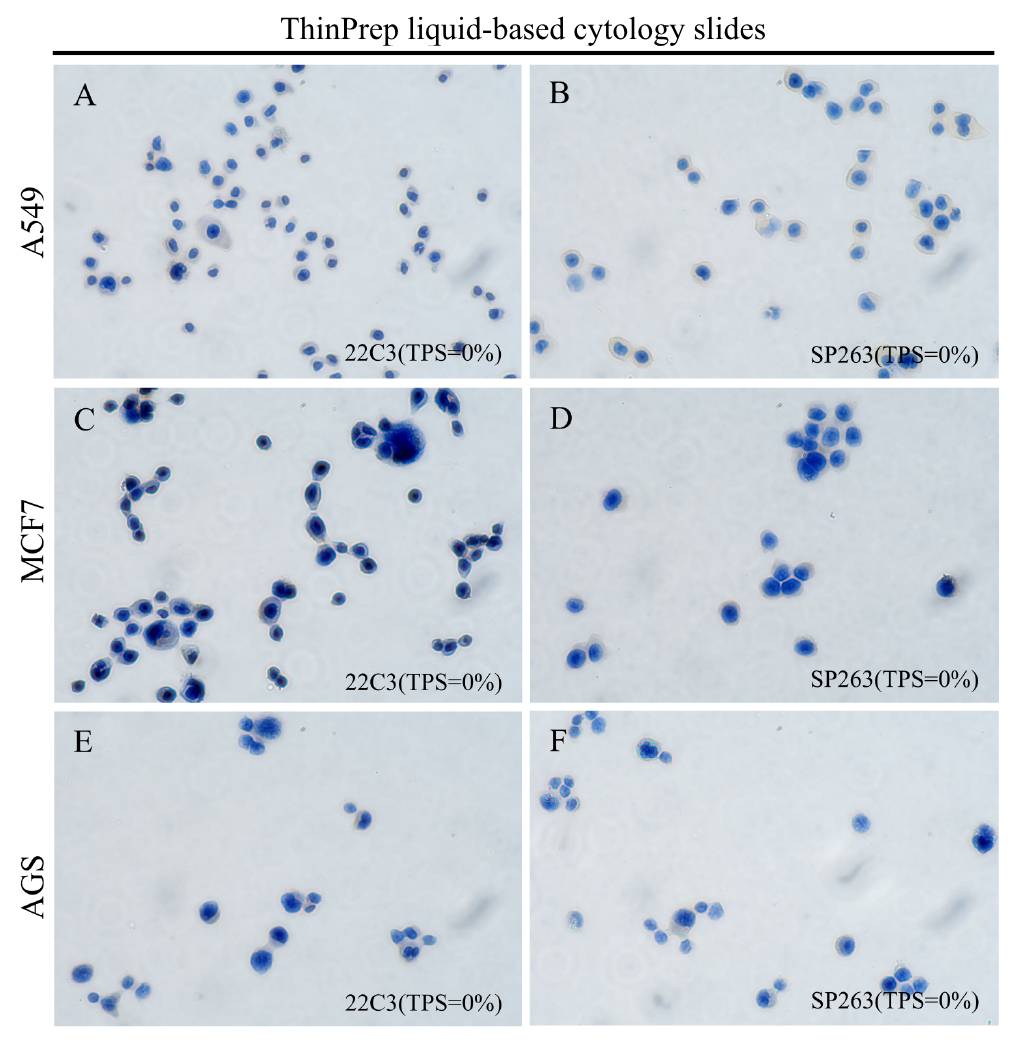


**Supplementary Figure S1.** Immunocytochemistry with clones 22C3 (Dako) and SP263 (Ventana) on ThinPrep liquid-based slides from three validated low-PD-L1-expression tumor cell lines (lung adenocarcinoma A549, breast cancer MCF7, and gastric cancer AGS) shows negative PD-L1 staining (×400).


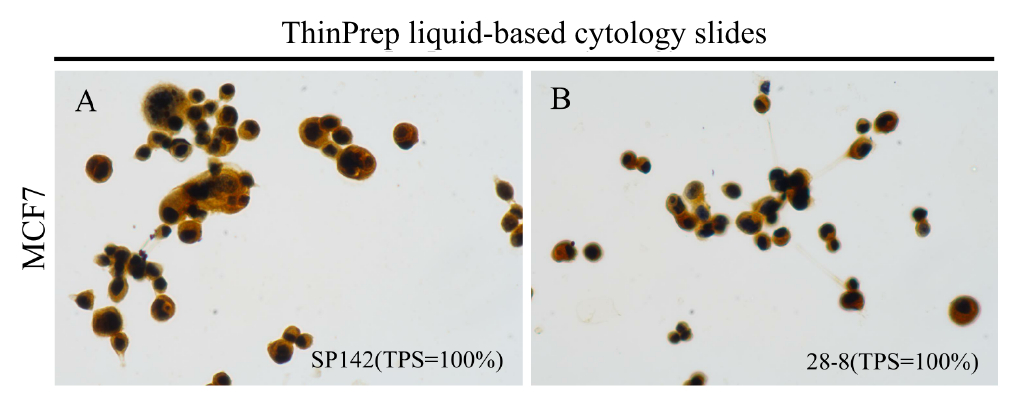


**Supplementary Figure S2.** Immunocytochemistry with clones 28-8 and SP142 (Abcam) on ThinPrep liquid-based slides from validated low-PD-L1 expression tumor cell line MCF7 displays false-positive PD-L1 staining (×400).


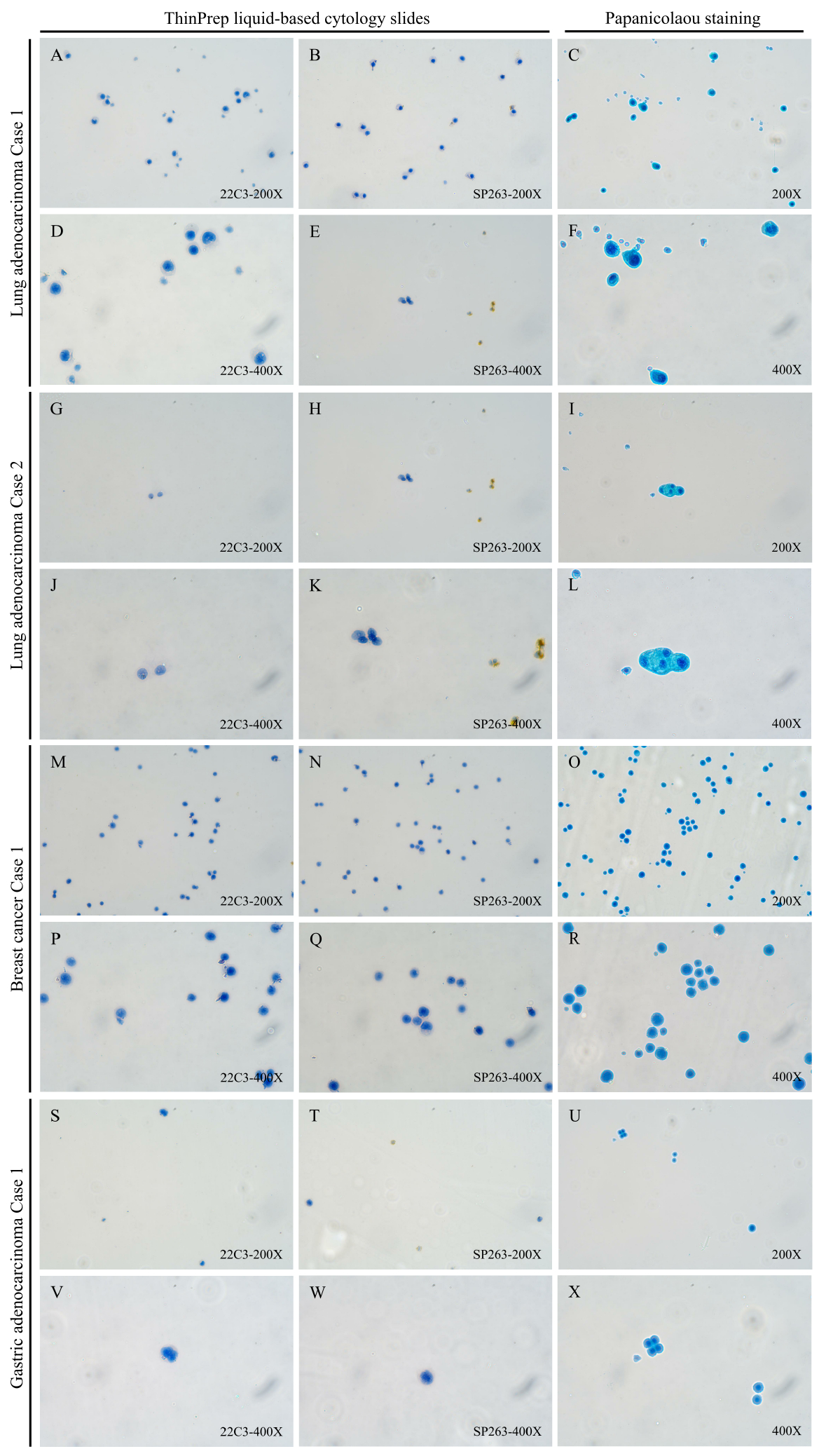


**Supplementary Figure S3.** PD-L1 immunocytochemistry with clones 22C3 (Dako) and SP263 (Ventana) on CSF ThinPrep liquid-based cytology slides from LM patients with various cancer types (×200 & ×400). **(A-F)** Detection results of the 22C3 and SP263 antibodies and papanicolaou staining in a PD-L1- negative lung adenocarcinoma case 1 (≥100 tumor cells); **(G-L)** Detection results of the 22C3 and SP263 antibodies and papanicolaou staining in a PD-L1-negative lung adenocarcinoma case 2 (<100 tumor cells); **(M-R)** Detection results of the 22C3 and SP263 antibodies and papanicolaou staining in a PD-L1-negative breast cancer case 1; **(S-X)** Detection results of the 22C3 and SP263 antibodies and papanicolaou staining in a PD-L1- negative gastric adenocarcinoma case 1.


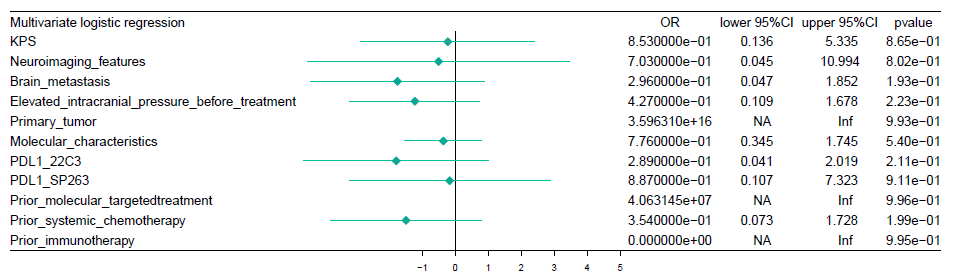


**Supplementary Figure S4.** Multivariate logistic regression analysis of variables for clinical response in 45 leptomeningeal metastasis patients receiving intrathecal immunotherapy. Abbreviations: KPS, Karnofsky Performance Status; PDL_22C3, PD-L1 immunocytochemistry with clone 22C3; PDL_SP263, PD-L1 immunocytochemistry with clone SP263.

# Supplementary Tables

See the two Excels uploaded separately.
